# The Polish Catholic Church in Martin Coronado, Buenos Aires, and its Role in the Life of the Argentinian Polish Community during the COVID-19 Pandemic

**DOI:** 10.1007/s10943-022-01701-7

**Published:** 2022-12-08

**Authors:** Kamila Ziółkowska-Weiss

**Affiliations:** Institute of Geography, Department of Tourism and Regional Studies, Pedagogical University of Krakow, Ul. Podchorążych 2 (Room 537), 30-084 Kraków, Poland

**Keywords:** Polonia, Buenos Aires, Martin Coronado, Argentina, Catholic Church, Bernardines

## Abstract

The objective of the article is to determine the role of the Catholic Church in the life of the Polish community in Argentina as exemplified by the Polish Catholic Centre in Martin Coronado during the pandemic caused by the SARS-Cov-2 coronavirus. The article presents opinions of the Polish community in Buenos Aires concerning the priestly service of the Bernardine friars during the pandemic. The research confirms that the Argentinian Polish community evaluates the work of the clergymen very highly and emphasises that thanks to their support, peace and constant contact through, inter alia, introduction of the broadcast of the masses in the Polish language, they felt the care and support of the priests, which was necessary during social isolation. The article will also present a proposal of the model of activities that Polish priests from Martin Coronado can implement during the COVID-19 pandemic so that the faithful feel even greater support and closeness to the Polish missionary centre in Argentina.

## Introduction

COVID-19 pandemic, passing over the world, resulted in the death of hundreds of thousands of lives globally, causing fear and a range of social, political and economic consequences (Krzewicki, 2020). Pandemics constitute a significant health problem in the world, with a major impact on the populations of affected countries. On 21 February 2020, 47 confirmed cases of COVID-19 were reported in Europe, including the first fatal case. On 25 February 2020, Brazil was the first country in South America to report a coronavirus disease. Within weeks, countries across the continent closed their borders and enforced blockades. On 14 April, Latin America registered more than 65,000 cases of COVID-19. Before the disease spread to Argentina, the Ministry of Health intensified surveillance mechanisms. Massive media campaigns aiming at providing the information and introducing the guidelines for people to be aware of the disease and its symptoms were launched with the above-mentioned measure.

COVID-19 reached Argentina 64 days after the first case was identified in China. The first officially confirmed case of SARS-CoV-2 in Argentina was a traveller who was diagnosed with the disease on 3 March. On 11 March, all travellers from the countries where COVID-19 cases were confirmed were asked to remain isolated for 14 days. Since then, the Argentinian government took a number of measures to mitigate its impact in this initial phase (Gemelli, 2020). Since March, the number of cases was insignificantly increasing; the first death due to COVID-19 was recorded in Argentina on 9 March. On the same day, a dedicated telephone line was included in the general consultation. As the number of cases increased in Argentina and worldwide, epidemiological controls were gradually intensified.

On 19 March, 16 days after the first case of COVID-19 was detected in Argentina, a decree that established a mandatory national quarantine until 31 March was promulgated by the former president. A total free movement of people was banned, allowing only some people to travel to their place of work. Flights were cancelled and borders were closed. On 25 March, 502 cases of infection were reported, with eight deaths. Schools and universities were closed on 16 March, moving towards the system of distance learning. Borders were closed almost immediately, except for flights bringing Argentines from abroad and vice versa for foreigners (Blackman et al., 2020).

At the end of June 2021, Argentinian authorities announced that a state of epidemic emergency would remain in the country until 31 December 2021. During the current quarantine phase in the provinces where the epidemiological situation has significantly worsened, restrictions on freedom of movement outside the immediate vicinity of the residence have been reinstated. Transport between provinces indicated by national authorities has been completely suspended. Decisions to restrict freedom of movement at night hours and to ban the organisation of events, the use of public transport without permits, have been taken as well as schools, churches and restaurants were closed or their activity was significantly limited. In the city of Buenos Aires, there is a curfew from midnight to 6:00 a.m. (status until 9 July 2021). Travelling between the Argentinian provinces is restricted—a mobile travel application and a medical certificate of the negative result of the COVID-19 test are required. The Argentinian land border was completely closed to passenger traffic until November 2021; entry and exit from Argentina were only possible by air if justified. Only the San Sebastian and Integracion Austral Road crossing that provides a link between Tierra del Fuego and the remaining part of the country has remained open.

At the end of 2021, Argentine society, polarised in many ways, is further divided between those who support the reinstatement of harsh measures to prevent further infections, and those who desperately demand the urgent need to return to work in order to maintain their social lives. The basic cohabitation agreements present a challenge that requires consensus in this critical situation (Cereghini & Gemelli, 2021).

The number of people confirmed to be infected with SARS-CoV-2 virus has exceeded 530.4 million worldwide (as of May 27, 2022). More than 6.3 million patients have died from COVID-19 since the beginning of the pandemic. The most affected regions of the world by the pandemic are Europe (over 195.9 million cases), Asia (over 155.1 million), and North America with over 101.1 million infections. Europe is the region where COVID-19 records the most deaths. 29.2 per cent of all coronavirus victims worldwide have died there. In Asia, fatal cases account for 22.7 per cent of all COVID-19-related deaths.

In Argentina, where 9,178,795 COVID-19 cases have been reported, 128,825 of them have died. Below are tables showing the number of COVID-19 cases, and deaths by continent and in selected countries, including both Argentina and Poland (www.worldometers.info).

Table 1 Continents, containing the number of COVID-19 cases, deaths, and recoveries.

**Table 1:** Continents, containing the number of COVID-19 cases, deaths, and recoveries

Continents	Containing the number of COVID-19 cases	Deaths	Recoveries
Europe	196,013,441	1,839,685	184,897,787
Asia	155,175,381	1,430,845	147,795,601
North America	101,194,906	1,473,007	95,655,013
South America	57,560,242	1,298,379	53,439,655
Africa	12,130,734	254,825	11,322,583
Australia and Oceania	8,603,383	12,160	8,114,152
Total	530,067,808	6,308,916	501,225,497

Table 2 Countries with the highest number of infections, including both data for Argentina and Poland. Source: www.worldometers.info [May 27, 2022].

**Table 2:** Countries with the highest number of infections, including both data for Argentina and Poland

Country	Containing the number of COVID-19 cases	Deaths
1. USA	85,628,581	1,030,958
2. India	43,148,500	524,539
3. Brazil	30, 921,145	666,319
4. France	29,439,416	148,129
5. Germany	26,254,124	139,132
6. United Kingdom	22,276,975	178,313
7. Russia	18,315,292	378,784
8. South Korea	18,053,287	24,103
9. Italy	17,355,119	166,476
10. Turkey	15,068,017	98,948
11. Spain	12,326,264	106,341
12. Vietnam	10,715,247	43,078
13. Argentina	9,178,795	128,825
14. Japan	8,737,523	30,458
15. Netherlands	8,081,992	22,314
20. Poland	6,006,809	116,305

Many authors in Poland have addressed the topic related to COVID-19, concerning both the situation in Poland and the world (Trepanowski & Drążkowski, 2022; Mróz, 2021; Mróz & Roszak, 2022; Domaradzki, 2021). However, so far there has been no topic dealing with the Polish community in Argentina, and the role of the Polish church during COVID-19 which makes this text innovative and pioneering among studies on missionary activities during the global COVID-19 pandemic.

Argentina, just after Brazil, is the second country in South America where the largest number of Poles, mainly emigrants, who were looking for better living conditions settled. Polish emigration to Argentina started in the middle of the nineteenth century. The Poles who settled in Argentina brought the customs, traditions and values from their homeland that are still cultivated by descendants of the Polish emigrants.

In Argentina, in the second decade of the twenty-first century, there are about 120,000 descendants of Poles living permanently (Report, 2012). The main Polish communities are located in the agglomeration of Buenos Aires and in the province of Misiones. Numerous groups of Poles also live in the cities of Rosario, Cordoba, Santa Fé, Wanda and Comodoro Rivadavia. About 16,000 descendants of Poles living in Argentina hold the Polish citizenship (Wasilewski, 2019).

The largest concentration of Poles in Argentina is in Buenos Aires, and as result, the choice of the research location was not accidental. In addition to the large Polish community in Buenos Aires, there is the Polish Catholic Centre in Martin Coronado that is the mainstay of the Polish community life in Argentina. According to the data obtained by the author during her research in the Polish Catholic Centre in Martin Coronado, it is estimated that currently above 1000 Polish families who had and still have contact with the Polish missionary centre during their emigration to Argentina live in Buenos Aires and Greater Buenos Aires.

Bringing up the topic of contemporary issues of the Polish pastoral ministry in Argentina, the information on this topic is primarily drawn from the studies of Friar A. H. Wróbel, (2002, 2004, 2020), Twaróg (2017) as well as from the studies of the Catholic Encyclopaedia (Kaczmarek, 1985).

The work of the Catholic Church in Argentina is mainly focused on religious care for emigrants. The newly arrived immigrants seek their first contact with the Polish society in the Polish Church. The forms of piety and faith brought from the homeland constitute a form of expression of the identity by Poles.

Polish emigrants also most often define their nationality through religion, which is often for many of them the only institution of social life, helping them to fully cross the borders of the country and giving them a sense of preserving their identity in a reality new to them. This was particularly noticeable in the former Polish patriotic emigrants of the nineteenth and twentieth centuries, who tried to organise their religious life in the places of their new settlement according to the customs they had brought from Poland. Their entire spiritual and cultural legacy, all the tradition in which they had grown up in their homeland, was present in their lives further abroad. A great role was played mainly by Polish pastors and Polish parishes, which supported the emigrants and their families in the development of religious life. Their religious life was also imbued with many elements of traditional Polish piety, having its basis in national culture. Today it is similar, especially with emigrants in the first period of settlement, who, not yet sufficiently familiar with the language of their new country, are particularly strongly attached to those forms of piety they brought with them from Poland. In this way they express their cultural-religious identity, which binds them to their memories of their homeland.

High rates, coupled with physical and social restrictions, have resulted in health, economic, and psychological uncertainty. Along with increased levels of anxiety, depression, and loneliness in the general population, COVID-19 has caused another global "pandemic"—loneliness and fear (Lahav, 2020; Salari et al., 2020). This is why the sense of security among the Argentine Polish community that they receive from the priests is so important and relevant. The COVID-19 pandemic has made spiritual care among the public as important as health care. Research conducted at the beginning of the pandemic showed that spiritual care during hospitalisation and a global pandemic plays an important role in creating a sense of security, and often survival (Busfield, 2020; Cadell et al., 2021; Drummond & Carey, 2020; Dutra & Rocha, 2021; Ferrell et al., 2020; Hamilton et al., 2021; Jones et al., 2020; Koenig, 2020; Carey et al., 2022; Murphy, 2020; Papadopoulos et al., 2021; Pirutinsky et al., 2020; Sarmiento, 2020; Shapiro et al., 2021; Sohail et al., 2020; Thomas & Barbato, 2020).

In an article by Llius Oviedo (2019), which was published just before the outbreak of the global pandemic, it can be read that religion is believed to give meaning or purpose to life for most of the world's population. However, it seems that the importance of religion and the role of the church during the pandemic played a larger role than before among the public around the world. Not only does the research presented in the article prove this, but one can find numerous articles in the literature in which researchers describe what role the church played among the public during the COVID-19 pandemic. Of particular note was Sereczynńka et al. (2021), in an international study among respondents from Spain, Italy, Poland, and Finland, that they conducted at the height of the pandemic, indicating the role and importance of religious and spiritual capital. The authors distinguish between these concepts and report that "religious capital", in their view, requires investment in an established religious institution through frequent participation and engagement, whereas "spiritual" capital" can be obtained through more informal means or private practice, such as personal study and meditation, in a more autonomous or unaffiliated manner. The authors examined questions related to meaning and coping in these unique circumstances, and assessing the impact of religious beliefs and practices during isolation. After administering 1162 survey questionnaires, the authors found that respondents clearly responded that their religious and priests during the pandemic found a way to communicate with them, which was especially meaningful to them. These data support the notion that one can do well in isolation when supported by a church or religious institution. The research collected by Sereczyńska et al. (2021) indicates that religious capital is clearly useful for coping in difficult and challenging times, which supports the thesis presented in this paper.

In contrast, in their study, Büssing et al. (2022) write about the weakening of faith among the German community. Their analysis showed that with the second wave of infection and its second blockade, confidence in the church, along with prayer and meditation, declined. Also, the surge in coronavirus-related illnesses was associated with a decline in well-being and continued loss of faith. They observed these changes in both Catholics and Protestants, as well as in younger and older people. In addition, long phases of uncertainty and social isolation, devoid of the meaningful support usually provided by religious communities, may also have challenged the ability to cope with religion of clergy themselves.

The Polish Catholic Centre of the Franciscan and Bernardine Friars in Martin Coronado (the province of Buenos Aires) was founded in 1958. Construction works that began in 1957 were managed by Friar Justinian Maciaszek who arrived in Argentina at the end of February 1949. On 7 June 1958, consecration of the newly built chapel and monastery took place which was a great event for the Polish community in Argentina. Besides Friar Maciaszek, an important role in the construction of the monastery and the chapel was played by Friar Andrzej Smoleń who came to Argentina from the USA in 1953. He continued the pastoral work in Martin Coronado in 1959 after the death of Friar Maciaszek. The Polish Catholic Centre in Martin Coronado is also called 'Maciaszkowo' after Friar Justinian Maciaszek, its founder and builder. The Polish Saturday School in Martin Coronado began its work with dedication of the chapel in 1958.

Thanks to ‘Schematism of the Polish clergy in Argentina’ we know that 82 Polish priests work in Argentina, including 4 of them in Martin Coronado. Among the priests providing missionary service in Argentina there are: Diocesan priests, Franciscans (Bernardines), Michaelites, Redemptorists, Missionaries of the Holy Family, Discalced Carmelites, Divine Word Missionaries, Salesians. There are also 29 nuns working in Argentina: Albertine sisters, Resurrection sisters, Sacred Heart sisters, Franciscan Missionaries of Mary, Grey Ursulines, Missionary Congregation of the Servants of the Holy Spirit, Servants of the Divine Mercy Gospel.

An important task of the Polish pastoral ministry in Argentina is to maintain and strengthen the ties between the Poles in Argentina, care for the elderly and Poles in need as well as promote the Polish language and culture. However, when the pandemic broke out, Polish priests working in Argentina faced new challenges and responsibilities caused by the global epidemic. Therefore, the objective of the article is to present the role of the Catholic Church in the life of the Polish community in Argentina as exemplified by the Polish Catholic Centre in Martin Coronado in Buenos Aires during the COVID-19 pandemic that posed an even greater challenge at that time than working and keeping up the spirits of the Polish compatriots on a daily basis.

The study will present the general results of the research conducted by the author among the Argentinian Polish community in Buenos Aires. Thanks to them, it was possible to familiarise with the role of the Catholic church in the life of the Polish community in Buenos Aires. Also, the statements of the Polish community (given to the author during the pandemic) in which particular attention was paid to the role and place of the church in their lives during the isolation and uncertainty caused by COVID-19 will be shown.

The undertaken research problem is important taking into account the extent to which the COVID-19 pandemic affected functioning of the faithful and participation in the life of the Poles in it. It is also significant due to the unfortunately still very dynamic development of the COVID-19 pandemic in Argentina. The presented possibilities of action by the clergy and their implementation may facilitate the functioning of the Argentinian Polish community during the pandemic and help them to maintain the sense of their Polish national identity. The presented research is the first of its type to demonstrate the role of the missionary clergyman performing priestly ministry among his compatriots outside Poland in a situation of epidemiological threat and social isolation. These results may be of great importance and their continuation seems an obvious procedure. Consequently, it can safely be said that this is precursory research in relation to the world space of clergy.

## Materials and Methods

In January and February 2020 (just before the outbreak of the SARS-CoV-2 pandemic), the author had the opportunity to visit Argentina and stay at the Polish Catholic Centre of the Franciscan Friars in Martin Coronado, Buenos Aires. Apart from the library search conducted in the collection gathered for years by the Bernardine Friars in Martin Coronado that is located within the walls of the monastery, the author conducted numerous talks and interviews among the Polish community whose main objective was to determine the role the Catholic Church in the life of the Polish community in Buenos Aires. Above all, while staying with Polish monks at the Polish Catholic Centre in Martin Coronado, the author took part in the participant observation analysing the manner the Polish clergy maintain the sense of the Polish national identity among the Polish community on a daily basis, their daily work and numerous responsibilities they have each day.

Even then, the author got to know the Polish community in BA, with whom she kept in touch, and in whom she gained trust. The relationship between the researcher and the participants is very important, affecting the collection, analysis, and interpretation of data. Therefore, when interviewing participants in the study, for many of them she was not an unknown anonymous person. The people with whom the Author conducted the research knew that she works at the university, that she conducts scientific research about Polish communities around the world, that she is a member in international research about the Polish community, and that the results obtained will only be used for aggregate analysis.

Thanks to constant contact with Polish missionaries working in Buenos Aires, after return to Poland and the outbreak of the pandemic, the author had complete and up-to-date information regarding the work of the clergy in the last months (from March 2020 to November 2021) and their significant role and support during the pandemic for the Polish community during their isolation and inability to function in the traditional, accustomed manner. She regularly wrote down the information obtained on a current basis and phone calls as well as she kept records, inter alia, by following the information posted on the website of Maciaszkowo and following the YouTube channel run by the Rector of the Polish Catholic Mission, Friar Jerzy Twaróg. Thanks to his kindness, the author obtained information regarding how their pastoral work has changed due to the global pandemic.

Due to the pandemic and impossibility of entering Argentina and the intention the continue the research, the author has decided to conduct interviews with the Argentinian Polish community through the Internet connections. A questionnaire interview, classified as qualitative research, constituted the method applied during the presented research. It was based on the questions previously prepared by the researcher whose aim was to obtain specific information through interrogation, i.e. asking about them (Sztabiński, 1997). It is worth mentioning that the quality of particular answers depends on the proper construction of the interview questionnaire. It applies to clear formulation of questions and their comprehensible transfer to the respondent.

During the pandemic, the author interviewed 20 people. Adequacy of the sample is crucial when evaluating qualitative research. The author used purposive sampling, people she had previously met, as this method is usually used to select participants who have special characteristics, and can provide rich, relevant and diverse data relevant to the research question. The author also used the "snowball sampling" method of asking participants to help the researcher identify other similar participants. This method is used when it is difficult to find a specific population of participants. Considering the fact that the Polish community in Argentina is a small community, and also one that knows each other well through shared cultural identity and participation in Polish events, the snowball sampling method is a qualitatively good method to use in this study.

Each interview lasted approximately 30 min and the conversations were recorded with the consent of the respondents. Afterwards, they were re-processed and analysed by the author. The study was conducted in accordance with the principles of the Declaration of Helsinki, and in accordance with local legislation and national guidelines for research involving human subjects, ethical approval was not required. All participants were informed of their right to opt out of the interview, the ability to stop the interview at any time during the interview, to withhold information regarding their personal situation, and that they would be guaranteed full anonymity in the study, which serves to show a general trend and will be used as an aggregate summary. All interviews were audio recorded and transcribed according to code to the respective author. Any emotion, intonation, silence or emphasis was also transcribed for analytical purposes. None of the participants asked for permission to transcribe the interviews.

During the empirical research, a categorised interview was used with prepared questions about the role of the church among the Polish community in Buenos Aires, especially during the COVID-19 pandemic. The author also asked respondents how long they had been in Argentina, whether they had been associated with the church since the beginning of their emigration, their nationality and age.

The interview was recorded on digital media (voice recording only). Each interviewee had their personal identity concealed by assigning a number, which the author uses to describe the research material in the form of selected quotes.

Selected answers in the form of quotations are presented in this article. The main goal of the interviews was to check and find out the opinion of Argentinian Poles concerning the manner the work of the clergy has changed during the pandemic and how helpful it turned out to be.

The biggest constraint during the desire to conduct the research was the total ban on entry to Argentina for those without Argentine citizenship from March 2020 to May 2022. The inability to conduct on-site interviews involved reaching people living in BA via internet connections. Before respondents agreed to be interviewed, the author wrote an email to Polish associations operating in BA asking them to reach out to people of Polish descent asking them for contact. Repeated attempts to contact these people by making phone calls writing emails ended with 20 people agreeing to be interviewed. Another limitation during the research was the lack among respondents, especially the elderly, of the necessary equipment in the form of a computer needed to conduct the survey. Often these individuals needed the help of others to connect and participate in the interview. The author realises that the interviews conducted among 20 people may be an insufficient number, but she believes that the results of the research are fairly conducted and the presentation is clear.

In order for qualitative research to be conducted well and reliably, certain criteria are used in such research. One of them is the COREQ (Consolidated criteria for Reporting Qualitative research) method of reporting qualitative research, which is mainly used in interviews. The COREQ report was developed by Tong et al. (2007), which consists of 32 criteria that go into the compilation of the COREQ checklist, which is designed to check the reliability of survey execution. In conducting the study, the author kept these guidelines in mind and applied them. The COREQ criteria for a well-conducted study include such elements as: description of the relationship with the participants (previous relationships, participants' knowledge of the interviewer, characteristics of the interviewer); selection of the research sample (sample size, persons accompanying the interviews), data collection (description of the interviews conducted, audio recording, duration of the interview, transcription of the data); analysis of the data and conclusions (coding extraction of topics, control of participants); reporting (presentation of quotes, data, conclusions, extraction of main topics). All these elements were applied and described in the article.

The author also hopes to be able to continue the field research in BA. The conducted research is the first on the evaluation of priests not only in Argentina but in South America. The author hopes that the information presented in the article contributes to the continuation of similar issues among other minority groups around the world among other researchers dealing with the role of the church in emigration.

### The Result

During field research conducted in January 2020 in Buenos Aires, the author conducted participant observation among the Argentinian Polish community due to which the role of the Catholic Church in the life of the Polish community in Buenos Aires (before the pandemic) was determined. She interviewed representatives of the Polish community (embassy staff, teachers in a Polish school, scouts, people who run a blog for the Polish community in BA). Although the main objective of this article is to present the importance of the role of the Church among the Polish community in Buenos Aires during the pandemic, it should be emphasised that the research conducted at that time confirmed that for most of the respondents, the Catholic Church plays a very important role in their lives. It provides them with religious care, a sense of security and helps to maintain contacts between compatriots living in Argentina.

Poles in Argentina are truly attached to the Catholic faith and Polish traditions. They willingly and often participate in religious celebrations of the national holidays and anniversaries, organised by the Polish Catholic Centre in Martin Coronado. Polish priests in Martin Coronado, Buenos Aires, contribute to strengthening the bonds between the Polish community in Argentina through their pastoral activities, organisation of celebrations of the Polish holidays and anniversaries as well as teaching in the Polish school, organisation of pilgrimages, celebrating daily Masses in the Polish language, organisation of the Polish community meetings and annual pastoral visits.

Polish missionaries working in Buenos Aires often play the role of an organiser of both religious and cultural life of the Polish community in Argentina. The role of a priest in exile is not limited to celebration of the masses and administration of the sacraments; however, it also applies to cultivation of the Polish customs and traditions. Therefore, pastoral care of the emigrants is based on several foundations: spiritual and pastoral assistance, support and assistance to the emigrants (handling their needs) and cultivating the Polish traditions and customs among the emigrants. Considering the role the Catholic Church plays in the life of the Polish community on a daily basis, the role of the Polish priest among the Polish community has a special significance and spiritual support for them, even more during the closure and months of social isolation caused by COVID-19.

When the pandemic broke out, the service of the Polish priests in Martin Coronado in Buenos Aires resulted in limitation of their work, their contact with the Polish community and their daily masses. Polish priests in Argentina, bearing in mind how close the Polish community is to the Polish centre and how much they are needed by the Polish society, conducted the broadcasts of the Holy Masses for the Polish community through websites on social networks and a channel on YouTube from the very beginning of the restrictions in Argentina, ban on movement and closure of churches. In this form, the Argentinian Poles were able to participate in the Polish Masses, blessing of food during Easter, Midnight Mass and in each religious event which was broadcast on the YouTube.

Internet contact with priests and possibility of virtual participation in the Polish mass were greatly appreciated by the faithful who emphasised their importance and gratitude to the Polish Catholic mission for opportunity to participate in Polish rites in the national, Polish language. During the course of the pandemic, in the autumn of 2020 and in the spring and summer 2021, the author conducted twenty conversations through internet links with representatives of the Polish community in Buenos Aires in order to find out what the church in Maciaszkowo means to them during the pandemic isolation and which role the faithful expect from the Polish church during the pandemic.

Interview transcriptions were coded, read, and analysed using thematic analysis (Guest et al., 2012).

Twenty individuals were interviewed (see: Table 3) average age: 58.95; range: 38–81 years; of which fifteen had dual Polish-Argentine citizenship, four had Argentine citizenship, and one person had only Polish citizenship. All interviews were conducted in Polish, although the interviewer indicated that the interview could be conducted in Spanish or English. Twelve of the interviewees were female and the remaining eight were male. All of the people who took the survey have lived in BA for at least 20 years, (average age: 45.15; range 21–68) and all of the respondents stated that they have been participating in the religious life of the Polish Catholic mission in Martin Coronado for at least 15 years (average age: 40.95; range 15–60).

**Table 3:** Participant demographics

Respondent’s code	Gender	Age	Nationality	Period of time of living in BA	Participation in the religious life of the Polish Catholic mission (period of time)
CF1	Female	47	Argentine	43	42
CM2	Male	38	Polish–Argentine	22	15
CF3	Female	65	Polish–Argentine	56	56
CF4	Female	48	Polish–Argentine	41	41
CM5	Male	58	Polish	28	18
CF6	Female	54	Polish–Argentine	40	40
CM7	Male	44	Polish–Argentine	21	16
CF8	Female	39	Polish–Argentine	39	Since born
CF9	Female	74	Polish–Argentine	49	37
CF10	Female	69	Argentine	38	34
CM11	Male	81	Argentine	68	About 50
CM12	Male	77	Polish–Argentine	54	About 45
CF13	Female	58	Polish–Argentine	58	40
CF14	Female	41	Polish–Argentine	41	41
CM15	Male	64	Polish–Argentine	About 45	About 45
CF16	Female	76	Argentine	About 60	About 40
CF17	Female	63	Polish–Argentine	54	40
CM18	Male	66	Polish–Argentine	66	About 60
CF19	Female	72	Polish–Argentine	45	45
CM20	Male	45	Polish–Argentine	35	35

The table below shows the demographics of the respondents including their age, gender, declared nationality, length of life in BA, and participation in religious life at the Polish Catholic mission. The main topic of discussion was the perception of the role of the spiritual care of the Polish church in BA during the pandemic, the spiritual need for closeness, and the evaluation of the work of Polish priests in Martin Coronado. All participants in the survey expressed great gratitude for the spiritual support of Polish priests during the pandemic, and emphasised how important it was for them to have contact with priests during their social isolation. All assured that they felt cared for by priests, confident of receiving help from them in a crisis situation (both material and spiritual), and felt a special unity with them during Sunday online Mass broadcasts.

Below there are selected quotes from the people interviewed by the author.

‘I didn't go to Polish mass every Sunday, I don't participate in religious ceremonies, but I often came there to borrow Polish books. In Maciaszkowo, the clergy have a library with Polish books. It is the only such place in Buenos Aires. In the pandemic, borrowing books was possible. I called in advance; a book was prepared for me and it was usually waiting for me somewhere left. I read a lot of books in the pandemic. If it had not been for the Polish library, I would not have had anything to read in Polish.’ (CM2).

‘We are very grateful that the fathers organised the broadcast of the mass in the Polish language and we were able to participate in it. For us, people in exile, participation in the Polish Mass has a special meaning, especially now, in the era of the pandemic, support of priests and their care is particularly necessary.’ (CF3).

‘Now, during the pandemic, I have become even more aware of the importance of the Polish church and the presence of the Polish priests in Buenos Aires to me’. (CF4).

‘The Polish Church and the work of the Polish priests has always been important to us here in Argentina. No one has expected that the world could ever be affected by the pandemic situation that we have been witnessing and participating in since March 2020. The situation we found ourselves in and the spiritual assistance we have received from the Polish priests in Buenos Aires made us even more aware of the important role they play and how much they unite us in the Polish community’. (CM5).

‘How many words of support and peace we receive from the Polish priests when we listen to them broadcasting the Polish masses! Their words are invaluable and awareness that they are there gives us the inner peace’. (CF8).

‘Thank you, Priests, for recording masses in the Polish languages for us, Poles, giving us homilies and giving us such a huge support throughout the year 2020. Despite the fact that we could not meet every week in a Polish church, the possibility to participate in the Polish mass celebrated in Argentina was extremely important, particularly in the times of isolation and this huge uncertainty’. (CF9).

‘I would like to thank for the fact that the Polish priests throughout the year kept the Maciaszkowo website up to date, which enabled us to feel the sense of the community, not just a parish community, but—above all—a minority community all the time. It is very important not to lose the Polish spirit in exile, which could easily happen during our mandatory isolation’. (CF10).

‘When the pandemic began St. John Paul II Nursing Home in Martin Coronado. where my sister resides was completely closed to visitors. I did not see my sister for over a year. Thanks to the priests who celebrate daily Mass there, I had information about what was happening to my sister and what physical condition she was in. This gave me peace of mind and reassurance’. (CM11).

‘The pandemic could have become the perfect reason for ceasing to attend the Sunday mass in the Polish language and identify with the Polish society. There was an excuse—the pandemic and isolation. Thanks to the work of our priests and their great efforts to give us the sense that they are there and they support us and pray for us, it was impossible to cut ourselves off from the spiritual world’. (CM12).

‘The priests have recorded masses for us in the Polish language, informed us about religious events through websites, however, they also reminded us of the patriotic Polish holidays and Polish traditions. We are very grateful to them for this. Thank you for your blessings, prayers and support’. (CF13).

‘I think that without the spiritual support of our Bernardine Friars, I would not have been able to cope in this pandemic and isolation’. (CF14).

‘Now, I have an even greater desire to attend every Sunday Mass. It does not matter that it is on the Internet, the important thing is that it is conducted in the Polish language by our priests’. (CM15).

‘Thank you so much for your spiritual care, I knew and had confidence that I could count on them, and that I would get help from them if necessary, including material help if I was left destitute and jobless because of COVID-19’. (CF16).

‘I felt anxiety because of COVID-19. In the first months of the pandemic I did not leave my house for fear of being infected. Every Sunday I went to Mass in Martin Coronado. When we were isolated and churches in Argentina were closed, I felt fear, depression, and uncertainty. Fortunately, the priests transmitted the Masses from my church, and I could attend the Sunday service in Polish. I thank them very much for that’. (CM18).

‘I’ve been a part of the Polish Catholic community in Martin Coronado for over 30 years now. The pandemic has shown me how important a role Polish priests play in my life, and how important their mission is for me. I think that without their spiritual help, I would not have been able to survive the pandemic’. (CM20).

Apart from pastoral activities, Polish clergy in exile has also been involved in educational activities. They take care of teaching native subjects to children and young people and maintaining the cultural and patriotic ties with Poland. Therefore, the pastoral ministry in Argentina is also active in the educational field. In 1961 the Millennium of the Baptism of Poland Polish Saturday School was established in the missionary centre of Martin Coronado where children and youth are taught their native subjects and prepared for the First Communion. Fathers and the teaching staff prepare academies on the occasion of national holidays.

In the interview conducted by the author in February 2020, Teresa Bąk-Uzarowicz, a principal of the Polish Saturday School, recalls that in the times when she attended the Polish School in the 1980s, there were more students (about 50) and more lessons were held than today. Back then, children studied the Polish language, Religion, Geography, History, Singing and they had Art classes. All the children spoke Polish very well, they were children of people born mostly in Poland who went to Argentina after the war. Nowadays, there are much fewer children, but in the last years more and more adults want to learn Polish, attending the classes in the Polish school. In a partial attempt to tackle the pandemic worldwide, governments have closed some or all of their educational institutions, leaving 69% of the world's population of pupils and students without access to face-to-face or remote education (https://www.argentina.gob.ar/documentos-consejo-federal-de-educacion/leyes-y-normativa-general, 2020).

According to the UNESCO Institute for Statistics, 3.1 million students out of the 14.2 million ones in Argentina (as of June 2021) have been left without tutoring in this period. The teaching staff at the Polish Saturday School functioning at Maciaszkowo have organised remote lessons for their students so that none of the students lost contact with the Polish language and permanent teacher care.

## Discussion

In March 2020, all churches in Argentina, including the Polish Catholic Centre in Martin Coronado, were closed. Churches in Argentina remained closed until the end of October 2020. Since November, it has been possible to celebrate the Holy Masses outside churches, in parks, church gardens, observing sanitary regimes, wearing face masks and maintaining an adequate distance of 2 m between people. The Masses could not be celebrated inside the building, only outside, in the open air. In November, cemeteries were opened, with possibility to attend the burial of the deceased, yet, without the return to traditional Masses in the cemeteries. The cemeteries close each day at 1 p.m. It should be noted that in Argentina, if the burial concerns a person who has died of COVID-19, people attending the funeral are not allowed to enter the chapel, they can only participate in the outdoor funeral by escorting the deceased to the grave, also without participation of a priest. As of 11 October 2021, in Argentina, it is not possible to attend the Masses in the building again as it was possible in February–May 2020- up to 15–20 people depending on the size of the room and per floor space. The daily evening Masses in the Basilica of Our Lady in Lujan and in the other Argentinian churches are celebrated with the doors closed, without the faithful present and the masses are broadcast through the YouTube channel.

The role of the priest in emigration is particularly important. Pastoral work among emigrants constitutes a great challenge; they need more pastoral care than they would normally receive if they lived in their native country. Polish emigrants very often relate to their nationality through faith and religion, which is a factor that maintains their national identity. Clergymen are faced with the task of supporting the families in emigration and, above all, of protecting them from new social and cultural trends by preserving the national memory. The Catholic Church in Argentina creates opportunities to deepen faith for the Polish community in Argentina, using their mother tongue, taking care to educate the descendants of Poles in the Polish spirit, according to the Polish traditions and customs. A large part of the Polish community in Buenos Aires consists of elderly people who came to Argentina in the 1940s and 1950s as small children. Today, they are in their 1960s and 1970s and they are particularly afraid of the consequences of the coronavirus disease if they become infected with it. Religious congregations have sought to maintain contact with the faithful, shifting toward online services, spiritual collections and retreats, community prayers, and sacraments (O'Brien, 2020). As a result, the possibility to participate in the Masses broadcast online by priests is particularly important and significant for them. This was particularly emphasised by those interviewed by the author (respondent’s code: CF:3; CM:15).

The pandemic situation presents the important role of the Polish priests in the mission for the Polish community in Buenos Aires.

It should be noted that the Franciscan Friars work at the cemetery in Pablo Podesta on a daily basis where they celebrate several funerals a day. They also carry out charity activities by commuting to the Ewa Peron Clinic in San Martin where they celebrate the Holy Masses and administer the sacrament of anointing of the sick every day. The pastoral service of the Franciscan Friars is not only conducted in Martin Coronado, they also go to: Llavallol, Merlo (the Holy Mass once a month), San Justo (the Holy Mass—4 times a year), San Jose, Burzaco (the Holy Mass in the Polish language—occasionally). In the period from October to December, a Polish Christmas-time pastoral visit takes place. Friars visit about 650–700 families in Greater Buenos Aires with Christmas wafers. They also implement Christmas-time pastoral visits among about 500 families in the province of Misiones (in Azara, Apostoles, Wanda, Lanuse). In 2020, Christmas-time pastoral visits in the homes of the faithful did not take place; however, Friars connected with the faithful through the Internet giving them blessings and listening to their parishioners.

Polish priests in Argentina through their pastoral activities, organising celebrations of Polish festivals and anniversaries, teaching in Polish schools, organising pilgrimages, celebrating daily Holy Masses in the Polish language, organising Polish meetings and annual pastoral visits contribute to strengthening the bonds between the Polish community in Argentina. Polish missionaries working in Buenos Aires often play the role of organisers of both religious and cultural life of the Polish community in Argentina. Therefore, the pastoral care of emigrants is based on several foundations: spiritual and pastoral assistance, support and help for emigrants (addressing their needs) and cultivating Polish traditions and customs among emigrants. Functioning of the Polish Catholic Centre in Martin Coronado in the conditions of the pandemic is not simple. The Franciscans undertake a number of initiatives and projects of a pastoral, socio-psychological, educational, cultural, patriotic or charitable nature, all of them for the Argentinian Polish community to allow the Poles to feel their support and assistance.

One of the initiatives taken by the Friars during the pandemic was introduction of television, radio and Internet broadcasts that were carried out from the very beginning of the closure of churches in Argentina. The new media helped the Church to stay in touch with the faithful during the pandemic and thanks to the immediate reaction of Polish priests to the epidemiological situation in Argentina, cooperation and contact between the faithful and priests was not disturbed. This was particularly emphasised by those interviewed by the author (respondent’s code: CF:8; CF:13; CM:18).

In Maciaszkowo, there is the Lieutenant Doctor Antoni Solowiej Museum of the Polish Army, the only one in South America, run by Friar Jerzy Twaróg. On 3 December 2017, celebrations were held for the 60th anniversary of the Polish Catholic Centre in Martin Coronado (Twaróg, 2018). Cooperation of the Friar Jerzy with the representatives of the Pilsudskites with a branch in Poland, Gen. Śliwa and Gen. Dr. Walentynowicz, resulted in signing the agreement between the two centres in 2020. On 16 February 2020, generals visited Maciaszkowo where—as representatives of the Pilsudski Association of Poland—presented numerous military appointments in Martin Coronado. This event undoubtedly demonstrates the great role of the Catholic Church in maintaining the sense of national identity of Poles in Argentina. Cultivation of the memory and national identity of Poles in exile and regular replenishment of the museum collections constitutes another patriotic activity, besides pastoral one, which is very important and crucial in exile. This was said by the person interviewed by the author (respondent’s code: CF:10).

In March, there were more than 150 Polish tourists within the territory of Argentina at the time of the introduction of restrictions in Argentina caused by CoV-2. The global chaos and inability to return to the country on a planned data have led to complete helplessness among tourists. Apart from Poles asking the Polish Consulate in Buenos Aires for assistance and shelter, many of them reported with this request to the Polish Missionary Centre in Martin Coronado. Thanks to many years of exemplary cooperation of the Polish Mission Centre with the Polish Consulate, with Ms Aleksandra Piątkowska, the Ambassador Extraordinary and Plenipotentiary of the Republic of Poland in the Argentine Republic, the Eastern Republic of Paraguay—all Poles who were in Argentina due to the pandemic could be safely brought to Poland.

Taking care of the elderly constitutes an important task of the Polish pastoral ministry in Argentina. On 29 August 1982, there was the opening of the St. John Paul II Nursing Home in Martin Coronado. The care of the Nursing Home is provided by the Albertine Sisters. Every day before the pandemic, fathers celebrated the Holy Mass there in the Polish and Spanish language. According to government guidelines, the facility was closed to any outsiders. During the pandemic, fathers very often acted as a bridge between the residents of the Nursing Home and their families who found out about the physical and mental condition of their loved ones through the sisters and the clergy. This was said by the person interviewed by the author (respondent code: CM:11).

In the Polish Catholic Centre in Martin Coronado, there is a large, catalogued library. It contains about 12,000 books and periodicals as well as the archives of the Bernardine Friars kept by Friar Dr Herkulan Antoni Wróbel. For years now, the Bernardine Friars have been helping students and doctoral students by making their collections available to them. Thanks to their assistance, several students wrote and defended their scientific theses. This way, the Bernardine Friars in the Polish Catholic Centre contribute to dissemination of knowledge about the Poles living in Argentina and their ancestors.

Due to the pandemic and the ban on traveling to Argentina from March 2020, and thus the possibility to conduct personal library searches on the spot in Buenos Aires, friars helped researchers in Poland dealing with the memory and cultivation of the Polish tradition in Argentina by scanning and sending fragments of books, articles and documents from the numerous collections in the Argentinean monastery to them, through multimedia technology. Also, thanks to running the Polish library by the priests, the possibility of reading Polish books among the Polish community in Buenos Aires during the months of social isolation met with great interest and willingness of their borrowing. This was said by the person interviewed by the author (respondent code: CM:2).

**Fig. 1:**
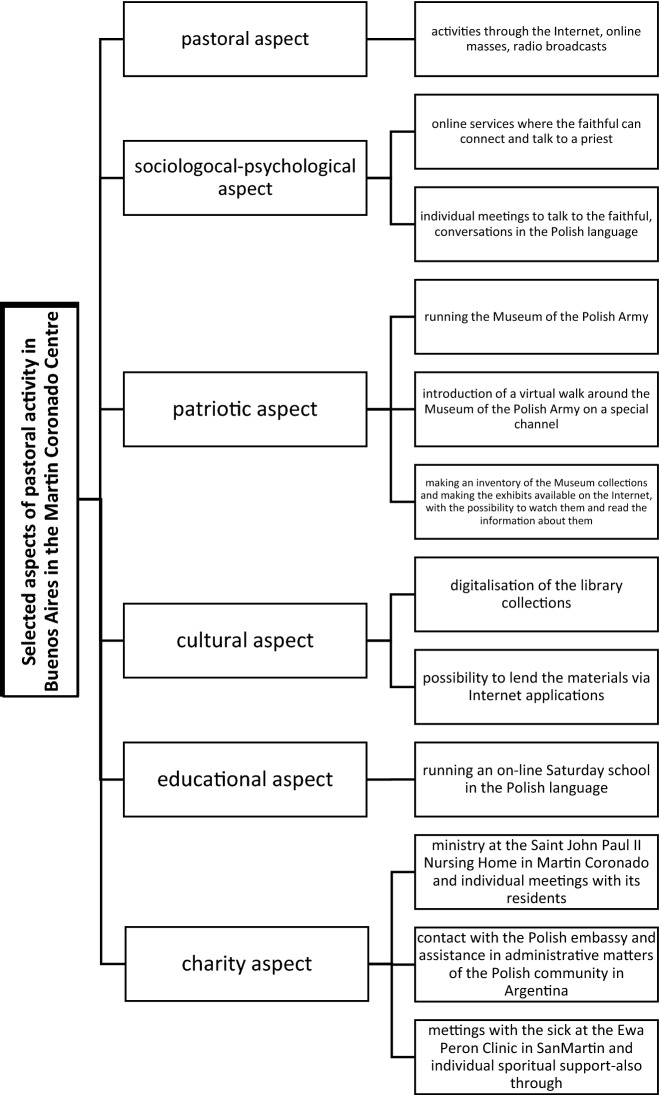
The model of religious, cultural and social activities undertaken by priests influencing the life of the Polish community in Buenos Aires during the COVID-19 pandemic. Source: author’s own compilation

The objective of the study is to indicate the activities and procedures that can be implemented by the clergy during the ongoing pandemic and the global epidemiological situation so that the faithful would not move away from the church and still feel a strong bond with the clergy (which is extremely important and necessary in the case of emigrants) due to the introduced restrictions in daily functioning (including the lack of possibility to participate in traditional services). Figure 1 presents the model of religious, cultural and social activities undertaken by priests influencing the life of the Polish community in Buenos Aires during the COVID-19 pandemic.

Therefore, the activities on the part of the clergy working at the Polish centre in Martin Coronado, Buenos Aires, thanks to which it will still be possible to maintain the bonds between a priest and a believer include:

- regular broadcasts of the Holy Masses in the Polish language available on the Internet channels,
- considering the fact that it is impossible to attend the services inside churches, it seems reasonable to develop the outdoor areas belonging to the parishes where it would be possible to celebrate the Mass for a greater number of people who can participate in the services. The weather in Buenos Aires is sunny for most of the calendar year and as a result, the proper development of the area around the church, by building outdoor altars, setting up benches for the faithful at a suitable distance and providing infrastructure, such as toilets, would—in the long term—allow to hold the outdoor Masses on a permanent basis with a greater number of people, without fear of introducing prohibitions and restrictions on the number of participants s in services;
- digitalisation of the library collection; possibility to borrow materials, books, articles in the Polish language, gathered in the library in Maciaszkowo using the Internet applications;
- making an inventory of the Museum collections and making the exhibits available on the Internet, with the possibility to watch them and read the information about them;
- introducing the possibility of participating in a virtual walk around the Museum of the Polish Army.

## Summary

The new outbreak of coronavirus (COVID-19) has disrupted all aspects of life around the world, with immediate and radical impacts on health and socio-economic situations (Oxholm et al., 2020). In response to the crisis, the United Nations introduced the concept of transition from the old to the new normal, emphasising the need to draw conclusions. In accordance with the international guidelines, Ministry of Education (MoE) of Argentina has introduced a series of ministerial resolutions on the pandemic (CEPAL, 2020). The suspension of pupils' participation in schools throughout the country and suspension of the public life are scheduled for the years 2020–2021.

The main objective of the article is to present the role of the Catholic Church on the Polish society in Argentina as exemplified by the missionary centre in Martin Coronado during the ongoing pandemic. As confirmed by the interviews conducted by the author among the Polish community in Argentina during the pandemic, the Catholic Church exerts a strong influence on the life of the Poles in Argentina and gives them great support, not only spiritual one, however, also the one in everyday life in the aspects concerning patriotism, education or cultural issues.

The outline of the activities of the Polish Church in Buenos Aires during the epidemic reveals an efficient and responsible handling of the emergency situation in which concern for the faithful and their safety was evident. Broadcasts of the Holy Masses were created for the faithful, taking into account their spiritual and religious needs. In Africa, Central America and South America, Internet broadcasts are used much more frequently than in Europe (Macula, 2019); however, participation in the Eucharist broadcast by mass media does not replace personal participation in the Holy Mass (Sambor, 2020). Until the outbreak of the coronavirus pandemic, broadcast Masses were addressed to the sick, the elderly and those immobilised by illness. However, this situation has changed dramatically with declaration of a general quarantine. Numerous parish and diocesan broadcasts emerged to respond to the growing interest and needs of the faithful in the emergency situation.

In the twenty-first century, in the era of new technologies and new means of digital communication, members of the Argentinian Polish community are rediscovering their roots. Thanks to encouragement of priests, they use widely available Internet tools and databases, create genealogical trees of their families, find their relatives, have up-to-date knowledge about changes taking place in Poland, learn about their existence and functioning of the Polish associations, parishes, folk groups, scout groups as well as other organisations and institutions gathering descendants of the Poles in Argentina.

The clergymen working among the Polish community in Argentina are engaged in helping those in need. When someone of the Poles living in Argentina asks them for help, they always try to help in a specific case. For many people of Polish origin who live in Argentina, the Polish Church and its surroundings is the first place they go to for help when they are in need, not only during the pandemic caused by COVID-19. However, a special situation in Argentina and rigorous restrictions in daily life that have been binding for many months, resulted in the fact that despite impossibility to gather on Sunday celebrations and other Polish meetings, the Argentinian Polish community can count on spiritual support and unity thanks to commitment of the Bernardine Friars.

Spiritual health and spiritual care given by priests in critical situations have long been recognised (Baldacchino, 2015; Fitch & Bartlett, 2019; Graves et al., 2002). As Domaradzki (2021) writes, the physical and psychological health consequences of SARS-CoV-2 are well described (Carfì et al., 2020; del Rio et al., 2020; Vindegaard & Benros, 2020), much less is known about spiritual needs and spiritual care during the COVID-19 pandemic. Therefore, research depicting what role clergy play during the pandemic is important and relevant also in the context of long-term emigrants. The studies showed that Polish priests working in Martin Coronado, Argentina, contributed to the sense of security among the Polish diaspora living in Buenos Aires.

The diagram presented in the discussion, which depicts the activities undertaken by the priests, that affected the life of the Polish community in Buenos Aires during the COVID-19 pandemic, touches on various aspects. It is not only the described pastoral aspect, and conducting regular Masses on the Internet, but it is also the support, and the sociological-psychological aspect consisting of individual meetings and conversations with the faithful, especially in Polish. The role of the Polish Catholic Church in Buenos Aires is also a patriotic aspect, especially necessary abroad. It is about emphasising the importance of maintaining Polish national identity, remembering Polish patriotic holidays and organising celebrations commemorating important for Poland historical events such as May 3rd (the adoption of the Polish Constitution) or November 11th (Independence Day). During Sunday Masses, when they directly reach a large number of people, pastors remind about Polishness during their sermons and emphasise patriotism among the faithful. Their role is not only to preach, celebrate and administer the sacraments, but also to take care of Polish customs and traditions by reminding their faithful about them, e.g. during religious ceremonies. Speaking about Poland, emphasising the origin of the faithful is an educational aspect, which contributes to passing Polishness from generation to generation. The pastoral role of the emigrants is based on several foundations: spiritual and pastoral support and assistance to the emigrants (addressing their needs), which in the case of the COVID-19 pandemic is of particular importance, and as the research has shown, the Argentinean Polish community needs this help, expects it, and most importantly, disinterestedly continues to experience it from the Polish priests working in Buenos Aires.
